# 

**Published:** 1893-12

**Authors:** 


					﻿PHYSICAL CULTURE, ITS ABUSE IN FOOT-BALL.
By ROBERT NEWMAN, M. D.
io cts. a copy.
DECEMBER, 1893.
$1.00 per* year.
HALLS®
jJoVRNALf
health
ESTABLISHED 1854
XL.
Wo. is!.
OFFICE. 23 PARK ROW.
Entered as Second-Class Matter at the Post-Office, New York.
				

